# Drug Use among Iranian Drivers Involved in Fatal Car Accidents

**DOI:** 10.3389/fpsyt.2014.00069

**Published:** 2014-08-29

**Authors:** Shervin Assari, Maryam Moghani Lankarani, Masoumeh Dejman, Marzieh Farnia, Ramin Alasvand, Mahmood Sehat, Mohsen Roshanpazooh, Mahmood Tavakoli, Firoozeh Jafari, Khodabakhsh Ahmadi

**Affiliations:** ^1^Health Behavior and Health Education, School of Public Health, University of Michigan, Ann Arbor, MI, USA; ^2^Center for Research on Ethnicity, Culture and Health, School of Public Health, University of Michigan, Ann Arbor, MI, USA; ^3^Social Determinants of Health Research Centre, University of Social Welfare and Rehabilitation Sciences, Tehran, Iran; ^4^Universal Network for Health Information Dissemination and Exchange (UNHIDE), Tehran, Iran; ^5^Medicine and Health Promotion Institute, Tehran, Iran; ^6^Health and Treatment Bureau of Iran Prisons Organization, Tehran, Iran; ^7^Substance Abuse and Dependence Research Center, University of Social Welfare and Rehabilitation Sciences, Tehran, Iran; ^8^Behavioral Sciences Research Center, Baqiyatallah University of Medical Sciences, Tehran, Iran

**Keywords:** substance use, drugged driving, Iran, drivers, driving, road accidents, injury

## Abstract

**Background:** Although the problem of substance use among drivers is not limited to certain parts of the world, most epidemiological reports on this topic have been published from industrial world.

**Aim:** To investigate pattern of drug use among Iranian drivers who were involved in fatal road accidents.

**Methods:** This study enrolled 51 Iranian adults who were involved in fatal vehicle accidents and were imprisoned thereafter. Data came from a national survey of drug abuse that was done among Iranian prisoners. The survey collected data at the entry to seven prisons in different regions of the country during a 4-month period in 2008. Self-reported lifetime, last year, and last month drug use was measured. Commercial substance screening tests were applied to detect recent substance use (opioids, cannabinoids, methamphetamines, and benzodiazepines).

**Results:** The commercial substance screening test showed three distinct patterns of recent illicit drug use: opioids (37.3%), cannabinoids (2.0%), opioids and cannabinoids (13.7%). 29.4% were also positive for benzodiazepines. The substance use screening test detected 23.5% of participants who had used drugs but did not disclose any substance use.

**Conclusion:** Opioids are the most common illicit drugs being used by Iranian drivers who are involved in fatal car accidents. The high rate of substance use prior to fatal car accidents in Iran advocates for the need for drug use control policies and programs as major strategies for injury prevention in Iran. There is also a need for substance screening among all drivers involved in fatal car accidents in Iran, as more than 20% of users may not disclose substance use.

## Introduction

Driving under the influence of drugs happens for several reasons. Recreational drug use may be prevalent among people who drive (1). Drugged driving happens if the driver is a drug user (2, 3). Drugged driving may also happen among professional drivers who drive long distances (4). Drivers who drive under the influence of drugs pose serious risk to others on the road (4, 5).

Although various drugs have different effects, most substances affect driving tasks, even in low dosages (6). An increased risk of crash may exist even in the absence of outward signs of impairment in the driver (4, 6). Drug use also increases the probability of poor outcomes following car accidents (7). It has been shown that risk of death following traffic accidents increases when it is secondary to substance use (7–9).

Prevention of drugged driving is an essential strategy for prevention of mortalities and morbidities due to unintentional injuries. Authorities, however, cannot design evidence-based policies and programs such as continued drug use education and screening programs if they do not have access to epidemiological information. Such information is essential for the design and implementation of policies that should be enforced to reduce traffic accidents attributed to drugs (4).

As the epidemiological pattern of substance use varies from one geographic location to another, knowledge of local epidemiological patterns of drugged driving is necessary for the improvement of road safety. Screening tests for substances in suspected cases are designed based on the epidemiological information about the type of drugs that are commonly used by drivers who are at high risk of accidents (10). For instance, in settings where opioids are not commonly used, suspected individuals for drugged driving undergo drug tests that do not cover opioids (1).

Besides the enormous economic burden of drugged driving and the fact that surveys on driving under the influence of substances have important implications for prevention (5), limited epidemiological knowledge exists on this problem in developing countries (1). Almost all published epidemiological knowledge in this field originates from North America, Europe, and Australia (2).

Different populations are being enrolled to the epidemiological studies on drugged driving (8, 9, 11–17). This includes general populations, drivers, professional drivers, and drivers who become involved in crashes (18, 19). One of the populations that is being studied in epidemiological surveys is drivers who are arrested or imprisoned following a fatal crash (20).

There is very limited information regarding drugged driving in Iran. Of the few studies that have provided epidemiological information on drugged driving (21–25), none have sampled drivers involved in fatal road accidents.

The main purpose of the current descriptive study was to investigate the problem of drugged driving in fatal car accidents in Iran. We also measured possible discrepancies between self-reported data and screening results for commonly used substances.

## Materials and Methods

### Design and setting

This national cross-sectional study was carried out by the Health and Treatment Bureau of the Iran Prisons Organization, Tehran, Iran. Data collection was conducted from September to December 2008.

An ethics committee approved our study protocol. Informed consent was received from all participants. Our participants were reassured that data would be kept confidential. All data were registered anonymously. No incentive was given to the participants.

### Participants and sampling

This study included 51 participants who were imprisoned because of motor vehicle accidents resulting in death. Participants were selected from 2,200 prisoners who participated in a national survey conducted in seven prisons in different regions of the country. Prisons were located at the following provinces: Tehran, Azarbayjan-e Sharghi, Golestan, Sistan and Balouchestan, Yazd, and Kermanshah. The study used a table of random numbers as the simple random strategy to include new prisoners.

### Measures

At the time of entry to prison, participants underwent a structured interview using a checklist. Interviews were conducted in private settings. All interviewers were men with master’s degrees in clinical psychology (the same gender as the participant).

Self-reported data on drug use during lifetime, last year, and previous month, were asked for the following drugs: cannabis, opium, opium derivates, powder heroin, compact heroin, Methamphetamine, cocaine, Lysergic Acid Diethylamide (LSD), and other drugs. Data were also collected on the mode of drug use, including: smoking, swallowing, sniffing, injecting, and inhaling.

### Drug screening

Two milliliters of blood were drawn from each participant using vacutainer tubes. The blood was contained in a preservative and was screened for drugs using enzyme-linked immunosorbent assay and gas chromatography–mass spectrometry analysis. Commercial screening tests were used for marijuana, meth, and opioids. These tests detect the presence of delta-9-tetrahydrocannabinol (THC), the active ingredient in marijuana, and of cannabis and methamphetamine (12, 26).

### Data analysis

We used SPSS for data analysis. Due to the low sample size, this study only provided descriptive statistics. We reported frequency tables for categorical variables. Means and standard deviations (SD) were reported for continuous variables.

## Results

### Descriptive statistics

All participants were men, and most were living in urban areas. Most participants were employed, married, and had not completed high school. Most participants did not have a history of previous imprisonment (Table 1).

**Table 1:** **Socio-demographic data among individuals who were imprisoned for fatal car accidents (*n* = 51)**.

	*n*	%
**LIVING PLACE**		
Urban	32	62.7
Rural	19	37.3
**EDUCATIONAL LEVEL**		
Illiterate	7	13.7
Read/write	2	3.9
Primary school	13	25.5
Guidance school	16	31.4
High school	3	5.9
Diploma	6	11.8
University	4	7.8
**MARITAL STATUS**		
Single	16	31.4
Married	34	66.7
Divorced	1	2.0
**EMPLOYMENT**		
Employed	47	92.2
Jobless	4	7.8
**PREVIOUS IMPRISONMENT**		
No	26	51.0
Once	18	35.3
Two times or more	7	13.7

Participants’ ages ranged from 21 to 56 years, with a mean (SD) of 32.4 ± 7.9 years. Monthly income ranged from 0 to $1,200, with a mean (SD) of $290 ± 211. Family size ranged from 1 to 14 persons with a mean (SD) of 4 ± 2 persons.

### Self-reported results

Based on self-reported data, the most frequently used drug during the last 30 days was opium (25%) followed by heroin or opium derivate (20%). Eight percent reported cannabis use. Although 4% reported alcohol use in their life time, none of the participants reported alcohol use over the past 30 days. The most common mode of drug use was smoking (81% of cases), followed by swallowing (17.5% of cases) (Table 2).

**Table 2:** **Self-reported history of drug use among Iranian adults imprisoned for car accident offenses (*n* = 51)**.

	Lifetime		Last year		Last month	
	*n*	%	*n*	%	*n*	%
**CANNABIS**						
No	41	80	45	88	47	92
Yes	10	20	6	12	4	8
**OPIUM**						
No	30	59	36	71	38	75
Yes	21	41	15	29	13	25
**METH**						
No	49	96	49	96	51	100
Yes	2	4	2	4	0	0
**ECSTASY**						
No	50	98	50	98	51	100
Yes	1	2	1	2	0	0
**HEROIN**						
No	35	69	38	75	41	80
Yes	16	31	13	25	10	20
**OPIUM DERIVATE**						
No	14	27	38	75	41	80
Yes	37	73	13	25	10	20
**OPIOIDS**						
No	24	47	27	53	30	59
Yes	27	53	24	47	21	41
**ANY DRUG**						
No	24	47	27	53	30	59
Yes	27	53	24	47	21	41
**ALCOHOL**						
No	49	96	51	100	51	100
Yes	2	4	0	0	0	0

### Drug screening

Drug tests were positive for opioids, cannabis, and both in 37.3, 2.0, and 13.7%, respectively. Of all participants, 29.4% tested positive for benzodiazepines. The drug screening test detected that 23.5% of the total sample were drug users who had not reported drug use (Table 3).

**Table 3:** **Drug test results among Iranian adults imprisoned for car accident offenses (*n* = 51)**.

	*n*	%
**OPIOIDS**		
Positive	26	51
Negative	25	49
**METH**		
Positive	0	0
Negative	51	100
**CANNABIS**		
Positive	8	16
Negative	43	84

Of the participants who had not reported drug use, 24% (*n* = 12) tested positive for illicit drugs. The false negative report of no drug use was seen for cannabis (10–12%), and opioids (6–14%), but not for Meth (0%) (Table 4).

**Table 4:** **Frequency of false negative of drug use among Iranian adults imprisoned for car accident offenses (*n* = 51)**.

	*n*	%
**OPIOIDS**		
Life time	3	6
Last year	5	10
Last month	7	14
**CANNABIS**		
Last month	6	12
Last year	5	10
Life time	5	10
**METH**		
Life time	0	0
Last year	0	0
Last month	0	0
**ANY DRUG**		
Last month	12	24
Last year	10	20
Lifetime	12	24

## Discussion

Based on our study, 60% of Iranian drivers who are involved in fatal car accidents use drugs. In the absence of drug screenings, 1/3 of the total number of drug users will be missed. As a result, investigation of drugged driving among Iranians who are involved in fatal car accidents should not merely rely on self-reported data. The most commonly used drugs among drivers involved in fatal crashes are opioids, followed by cannabis.

The high rate of drug use among Iranians who are imprisoned for fatal crashes can be explained by the known association between driving under the influence of drugs and the incidence and intensity of car accidents (9). Drugs impair mental function and reduce attention and concentration on driving tasks. In general, use of substances during driving may impair coordination, increase reaction time, and alter judgment of distance and speed. Drug use results in distortion of time, place, and space. Other effects of drug use are poor vision and muscle weakness. All these consequences of drug use increase risk and severity of traffic accidents (4, 6).

We could not find any published information about the epidemiology of drug use among drivers who were involved in fatal crashes in Iran. Most of the available knowledge in Iran is about the pattern of illicit drug use among drivers. In one study, 0.42% of applicants for driving licenses in Iran had positive opioid test results (21). In another study in Kerman, Iran, between 14.6 and 26.5% of drivers were opium addicts (23, 24). Kerman has one of the highest prevalence of opium use in Iran (27, 28).

A major part of the literature has enrolled drivers who were suspected of driving under the influence of drugs (1, 29–32). There are also few international studies on individuals involved in car accidents (12, 20). In Australia, cannabis (46.7%) was the most commonly found drug in injured drivers involved in motor vehicle collisions. The second most prevalent substance was benzodiazepines (15.6%), followed by opiates (11%), amphetamines (4.1%) methadone (3%), and cocaine (1.4%) (33). In Australia, 2.7% of individuals involved in car accidents reported their use of cannabis before the crash (33). In Hong Kong, 10% of samples tested positive for drugs. (34).

Of the drivers killed as a result of road accidents, 48% tested positive for alcohol or drugs. From those drivers who tested positive, 27% were positive for alcohol only; 19% for cannabis only; 28% for alcohol and cannabis (but no other drug); and 25% for a combination of drugs, including the combination of alcohol and/or cannabis. Of all deceased drivers who were positive for a combination of drugs (other than alcohol or cannabis), 23% were positive for opioids; 31% were positive for benzodiazepines; and 42% were positive for methamphetamines (35).

In contrast to our study, in many industrial countries, marijuana and alcohol are the most prevalent substances found among impaired drivers who are involved in fatal or non-fatal vehicle crashes. Other illegal drugs such as cocaine, opiates, and amphetamines may have lower prevalence in industrial countries (3). In Iran, opioids are the most commonly found drug among these individuals.

The pattern of drug use in subgroups of a community is affected by the epidemiology of drug use in the general population. We believe that this is not an exception for the case of driving under the influence of drugs (36–41). In most western countries, marijuana and alcohol are the most prevalent drugs used by the general population (42). In Iran, the pattern of risk associated with drug use is different from western countries (43–49).

The most commonly used drugs in the general population of Iran are opioids (50). In Iran, three of four illicit drug users use opioids (51). In Iran, opium may be used for self-medication (52). Iran has a higher rate of opium use in comparison with other countries (53). Iran accounts for about 85% of worldwide seizures of opium and more than 30% of worldwide seizures of heroin and morphine (54). Traditionally, opioids have a long history for recreational and medical use in Iran. Geographic location of Iran has posed Iran to such risk, as Iran is on the main opium trade route from Afghanistan to Europe (55).

Regarding the common modes of drug use, smoking, and eating were the most common in our sample. This was expected, as smoking is the most frequent mode of opioid use by the Iranian general population (50). The same finding has been reported among professional drivers. Based on a recent national study, opioids (46.8%) and heroin (27.6%) were the two most common drugs used by Iranian professional drivers (56).

Based on our study, only 4% of individuals involved in fatal vehicle accidents report alcohol use. This rate was <1% among professional drivers who used drugs in Iran (56). Based on some, but not all reports, alcohol is not a commonly used substance in Iran (57). This might be partially due to the fact that alcohol is banned in the country. Over 98% of Iranians are Muslim, and Islamic instructions ban its use (58–60). There are also other parts of the world where alcohol is not a common substance used by drivers (5, 35).

Our findings may have important public health implications for reducing unintentional injury in Iran. Iran is a developing country with a high incidence of fatal road accidents compared to other societies (22, 61, 62). The rate of deaths due to road traffic accidents in Iran is also higher than the global average (22). Such high fatalities have been partly attributed to the high rate of drugged driving in this country (23).

The current study advocates for the design and implementation of road side drug screening. Such drug testing can be routinely implemented at police stations. Random screenings may be implemented. There is also a need for a revision in current policies regarding charges associated with drugged driving. More restrictive regulations may contribute to the prohibition of driving under the influence of drugs and may promote traffic safety in Iran.

Post-crash drug screening of drivers is not routine in several countries around the world. In several industrial countries, drug analysis is not conducted unless there is a request by a police officer. We believe that universal drug screening of drivers who are involved in fatal motor vehicle collisions is needed in Iran. We also believe that tests should cover opioids and cannabis use, while alcohol may not be a major problem.

Countries that do not implement drug screening tests may have high rates of drugged driving (33). An important component of road safety efforts is anti-substance driving policies. Such policies may decrease the societal burden attributed to drug use by motor vehicle drivers (63, 64). Drugs covered by the screening tests should be tailored to each country. The findings of this study on the pattern of drug use among Iranian drivers who have been involved in fatal accidents may help Iranian policy makers with designing and implementing such screening tests. Our study findings advocate for the universal screening of all fatal accidents, or at least selected screening of suspected drivers.

The disturbingly high rate of drug use among individuals who are involved in fatal car accidents in Iran is an alarm and requires further initiatives to prevent fatalities due to drug related motor vehicle accidents. Based on our results, drug impaired driving laws in Iran should be revised in several ways. New policies will empower police force to detect drivers that are impaired by drugs.

Our findings highlight the need for various preventive measures to enhance road safety in Iran. Health education programs of drivers should include persuasive communication messages that minimize the rate of substance use among drivers. For teen drivers, education by parents is key and may be effective (65, 66). These interventional programs should emphasize common licit and illicit substances that influence driving performance and result in accidents and fatalities in each country (67). Intervention studies may test the efficacy of programs in changing driving behaviors and associated attitudes and beliefs among drivers (65, 66). Education material needs to be included in the education system and practiced from the school age. Education campaigns should also use public and mass media for education of the public. Strict regulations for issuing and renewing driver’s licenses may also lower rates of drugged driving and prevent fatalities associated with road traffic accidents (56).

The current study had several limitations. The data were not updated, as data were collected in 2008. The sample size was also small. The results may not be representative of all drivers or fatal road accidents in Iran. Data were not available on professional drivers who drive for a living or they were merely vehicle operators. Also, we did not know the location of the accidents (metropolitan areas or high-speed inter-city highways). These limitations were present because the main study was not designed specifically for the investigation of the pattern of drug use among drivers who were involved in fatal accidents, but had instead aimed to investigate drug use among Iranian prisoners. The current study used only a subsample of the participants of the main study in whom reason for imprisonment was a fatal road accident. The study, however, had a few strengths, as well. This was one of very few studies on the pattern of drug use in fatal accidents in Iran. The study did not solely rely on self-reported data, as it also implemented screening tests. Further studies with large and random sample sizes are needed. Published guidelines should be consulted for future drugged driving research (12).

## Conclusion

In Iran, relying on self-reported data will result in under-estimation of contribution of drugged driving to the fatal road accidents in Iran. Thus, drug screening tests should be implemented for all, or at least suspected cases, of drugged driving. Opioids and cannabis should be included in drug screening protocols of Iranian drivers.

## Conflict of Interest Statement

The authors declare that the research was conducted in the absence of any commercial or financial relationships that could be construed as a potential conflict of interest.

## References

[1] Calafat A Blay N Juan M Adrover D Bellis MA Hughes K Traffic risk behaviors at nightlife: drinking, taking drugs, driving, and use of public transport by young people. Traffic Inj Prev (2009) 10(2):162–9 10.1080/15389580802597054 19333829

[2] Substance Abuse and Mental Health Services Administration. 2008 National Survey on Drug Use and Health. Rockville, MD: Office of Applied Studies (2009).

[3] Soderstrom CA Dischinger PC Kerns TJ Kufera JA Scalea TM Epidemic increases in cocaine and opiate use by trauma center patients: documentation with a large clinical toxicology database. J Trauma (2001) 51:557–64 10.1097/00005373-200109000-00024 11535910

[4] National Highway Traffic Safety Administration. Results of the 2007 National Roadside Survey of Alcohol and Drug Use by Drivers. U.S. Department of Transportation Report No. DOT HS 811 175. Washington, DC: National Highway Traffic Safety Administration (2007)

[5] Beirness DJ Beasley EE A roadside survey of alcohol and drug use among drivers in British Columbia. Traffic Inj Prev (2010) 11(3):215–21 10.1080/15389581003735626 20544564

[6] Ogden EJ Moskowitz H Effects of alcohol and other drugs on driver performance. Traffic Inj Prev (2004) 5(3):185–98 10.1080/15389580490465201 15276919

[7] Lillsunde P Langel K Blencowe T Kivioja A Karjalainen K Lehtonen L Psychoactive drugs and accident risk in road traffic. Duodecim (2012) 128(18):1877–86 23088001

[8] Poulsen H Alcohol and Other Drug use in New Zealand Drivers, 2004–2009. Wellington, New Zealand: New Zealand Police

[9] Mason AP McBay AJ Ethanol, marijuana, and other drug use in 600 drivers killed in single-vehicle crashes in North Carolina, 1978–1981. J Forensic Sci (1984) 29:987–1026 6502125

[10] Dougherty J Prevalence of Drugs in ACT Drivers. Available from: http://www.tams.act.gov.au/_data/assets/pdf_file/0008/108791/Drugs_in_ACT_Drivers.pdf

[11] Walsh JM Flegel R Cangianelli LA Atkins R Soderstrom CA Kerns TJ Epidemiology of alcohol and other drug use among motor vehicle crash victims admitted to a trauma center. Traffic Inj Prev (2004) 5(3):254–60 10.1080/15389580490465319 15276926

[12] Walsh JM Verstraete AG Huestis MA Mørland J Guidelines for research on drugged driving. Addiction (2008) 103(8):1258–68 10.1111/j.1360-0443.2008.02277.x 18855814 PMC2690607

[13] Kelly E Darke S Ross J A review of drug use and driving: epidemiology, impairment, risk factors and risk perceptions. Drug Alcohol Rev (2004) 23:319–44 10.1080/09595230412331289482 15370012

[14] Christophersen AS Mørland J Frequent detection of benzodiazepines in drugged drivers in Norway. Traffic Inj Prev (2008) 9(2):98–104 10.1080/15389580701869190 18398771

[15] Silva OA Greve JMD Yonamine M Leyton V Drug use by truck drivers in Brazil. Drug-Educ Prev Polic (2003) 10(2):135–9 10.1080/0968763021000057727

[16] Soderstrom CA Dischinger PC Kerns TJ Trifillis AL Marijuana and other drug use among automobile and motorcycle drivers treated at a trauma center. Accid Anal Prev (1995) 27(1):131–5 10.1016/0001-4575(94)00043-L 7718074

[17] McCree DH Cosgrove S Stratford D Valway S Keller N Vega-Hernandez J Sexual and drug use risk behaviors of long-haul truck drivers and their commercial sex contacts in New Mexico. Public Health Rep (2010) 125(1): 52–60 20402196 10.1177/003335491012500108 PMC2789816

[18] Impinen A Rahkonen O Karjalainen K Lintonen T Lillsunde P Ostamo A Substance use as a predictor of driving under the influence (DUI) rearrests a 15-year retrospective study. Traffic Inj Prev (2009) 10(3):220–6 10.1080/15389580902822725 19452362

[19] International Transport Forum. Drug use among drivers. OECD Trans (2010) 9(17):22–38

[20] Li G Brady JE Chen Q Drug use and fatal motor vehicle crashes: a case-control study. Accid Anal Prev (2013) 60:205–10 10.1016/j.aap.2013.09.001 24076302

[21] Jahani MR Motavallian SA Kashani GHA Addiction in applicants of driving license. Kowsar Med J (2000) 5:289–29310689566

[22] Montazeri A Road-traffic-related mortality in Iran: a descriptive study. Public Health (2004) 118:110–3 10.1016/S0033-3506(03)00173-2 15037040

[23] Motevalian SA Jahani M Mahmoodi M Driving under influence of opiates in heavy vehicle drivers of Iran in 2001. Hakim (2004) 7:1–8

[24] Rajabizade G Ramezani MA Shakibi MR Prevalence of opium addiction in Iranian drivers 2001–2003. J Med Sci (2004) 4:210–3

[25] Jahani MR Roohollahi G Gharavi MJ Splenic hydatid cysts in a 20-year-old soldier. Mil Med (2004) 169(1):77–8 14964508 10.7205/milmed.169.1.77

[26] Couper FJ Pemberton M Jarvis A Hughes M Logan BK Prevalence of drug use in commercial tractor-trailer drivers. J Forensic Sci (2002) 47(3):562–7 12051337

[27] Ziaaddini H Ziaaddini MR The household survey of drug abuse in Kerman, Iran. J Appl Sci (2005) 5:380–2 10.1016/j.puhe.2012.08.011 23200101

[28] Shokoohi M Baneshi MR Haghdoost AA Size estimation of groups at high risk of HIV/AIDS using network scale up in Kerman, Iran. Int J Prev Med (2012) 3(7):471–6 22891148 PMC3415187

[29] Keller T Keller A Tutsch-Bauer E Monticelli F Driving under the influence of drugs and alcohol in Salzburg and Upper Austria during the years 2003-2007. Leg Med (Tokyo) (2009) 11(Suppl 1):S98–9 10.1016/j.legalmed.2009.01.059 19282219

[30] Siliquini R Chiadò Piat S Gianino MM Renga G Drivers involved in road traffic accidents in Piedmont region: psychoactive substances consumption. J Prev Med Hyg (2007) 48(4):123–8 18557306

[31] Siliquini R Piat SC Alonso F Druart A Kedzia M Mollica A A European study on alcohol and drug use among young drivers: the TEND by Night study design and methodology. BMC Public Health (2010) 10:205 10.1186/1471-2458-10-205 20420663 PMC2873581

[32] Siliquini R Bert F Alonso F Berchialla P Colombo A Druart A Correlation between driving-related skill and alcohol use in young-adults from six European countries: the TEN-D by night project. BMC Public Health (2011) 11:526 10.1186/1471-2458-11-526 21722358 PMC3145590

[33] Ch’ng CW Fitzgerald M Gerostamoulos J Cameron P Bui D Drummer OH Drug use in motor vehicle drivers presenting to an Australian, adult major trauma centre. Emerg Med Australas (2007) 19(4):359–65 10.1111/j.1742-6723.2007.00958.x 17655640

[34] Wong OF Tsui KL Lam TS Sze NN Wong SC Lau FL Prevalence of drugged drivers among non-fatal driver casualties presenting to a trauma centre in Hong Kong. Hong Kong Med J (2010) 16(4):246–51 20683065

[35] WHO. Alcohol and Injuries Emergency Department Studies in an International Perspective. Available from: http://www.who.int/substance_abuse/msbalcinuries.pdf

[36] Wesley RL Pattern of drug abuse. N C Med J (1983) 44(6):400 6576239

[37] Gossop M Drug dependence – 1. The pattern of drug abuse. Nurs Times (1978) 74(24):996–8 248701

[38] Narenjiha. Rapid Situation Assessment (RSA) of Drug Abuse in Iran 1998–1999. Ministry of Health, Tehran: I.R. of Iran

[39] Rahimi-Movaghar A Mohammad K Razzaghi EM Trend of drug abuse situation in Iran: Three-decade survey. Hakim (2002) 5(3):171–82

[40] Narenjiha H Rafiey H Baghestani AH Rapid Situation Assessment of Drug Abuse and Drug Dependence in Iran. Tehran: Darius Institute (2005).

[41] Razzaghi EM Movaghar AR Green TC Khoshnood K Profiles of risk: a qualitative study of injecting drug users in Tehran, Iran. Harm Reduct J (2006) 18(3):12 10.1186/1477-7517-3-12 16545137 PMC1431517

[42] Soderstrom CA Dischinger PC Kerns TJ Kufera JA Mitchell KA Scalea TM Epidemic increases in cocaine and opiate use by trauma center patients: documentation with a large clinical toxicology database. J Trauma (2001) 51:557–64 10.1097/00005373-200109000-00024 11535910

[43] Daneshmandan N Narenjiha H Tehrani K Assari S Khoddami-Vishteh HR Initiation to the first drug use among substance-dependent persons in Iran. Subst Use Misuse (2011) 46(9):1124–41 10.3109/10826084.2010.490971 21345165

[44] Rafiey H Narenjiha H Shirinbayan P Noori R Javadipour M Roshanpajouh M Needle and syringe sharing among Iranian drug injectors. Harm Reduct J (2009) 6:21 10.1186/1477-7517-6-21 19643014 PMC2731095

[45] Angoorani H Narenjiha H Tayyebi B Ghassabian A Ahmadi G Assari S Amphetamine use and its associated factors in body builders: a study from Tehran, Iran. Arch Med Sci (2012) 8(2):362–7 10.5114/aoms.2012.28566 22662012 PMC3361051

[46] Karimi M Ghaheri H Assari S Ahmadi K Moghani Lankarani M Moghani Lankarani R Drug injection to sites other than arm: a study of Iranian heroin injectors. Front Psychiatry (2014) 5:23 10.3389/fpsyt.2014.00023 24778621 PMC3985009

[47] Assari S Yarmohmmadi Vasel M Tavakoli M Sehat M Jafari F Narenjiha H Inconsistent condom use among Iranian male drug injectors. Front Psychiatry (2014) 4:181 10.3389/fpsyt.2013.00181 24772093 PMC3983495

[48] Ahmadi K Rezazade M Nafarie M Moazen B Yarmohmmadi Vasel M Assari S Unprotected sex with injecting drug users among Iranian female sex workers: unhide HIV risk study. AIDS Res Treat (2012) 2012:651070 10.1155/2012/651070 22506107 PMC3313628

[49] Mirabi P Vasel MY Moazen B Sehat M Rezazadeh M Ahmadi K Unprotected anal intercourse among Iranian intra-venous drug users. Front Public Health (2013) 1:34 10.3389/fpubh.2013.00034 24350203 PMC3859981

[50] Ahmadi J Pridmore S Alimi A Cheraghi A Arad A Parsaeyan H Epidemiology of opium use in the general population. Am J Drug Alcohol Abuse (2007) 33:483–91 10.1080/00952990701301293 17613976

[51] Day C Nassirimanesh B Shakeshaft A Dolan K Patterns of drug use among a sample of drug users and injecting drug users attending a general practice in Iran. Harm Reduct J (2006) 3:2 10.1186/1477-7517-3-2 16433914 PMC1397809

[52] Masoodi M Zali MR Ehsani-Ardakani MJ Mohammad-Alizadeh AH Aiassofi K Aghazadeh R Abdominal pain due to lead-contaminated opium: a new source of inorganic lead poisoning in Iran. Arch Iran Med (2006) 9(1):72–5 16649384

[53] UNODC. World Drug Report 2006. Available from: www.unodc.org/pdf/WDR_2006/wdr2006_volume1.pdf

[54] Emran M Razzaghi EM Afarin Rahimi M Hosseni M Madani S Prevention department, State Welfare Organization Ministry of Health, Tehran, I.R. of Iran RAPID SITUATION ASSESSMENT (RSA) OF DRUG ABUSE IN IRAN (1998-1999). Available from: http://www.unrol.org/files/RSA2000SUMMARY[1].pdf

[55] Gibson A Degenhardt L Topp L Global and Australian Heroin Markets. Sydney: National Drug and Alcohol Research Centre, University of New South Wales (2003).

[56] Narenjiha H Rafiey H Jahani MR Assari S Moharamzad Y Roshanpazooh M Substance-dependent professional drivers in Iran: a descriptive study. Traffic Inj Prev (2009) 10(3):227–30 10.1080/15389580902849017 19452363

[57] World Health Organization. Regional Office for the Eastern Mediterranean Questionnaire for Regional Situation Analysis on Drug Abuse 2003. Cairo: WHO Regional Office for the Eastern Mediterranean (2003).

[58] Mokri A Brief overview of the status of drug abuse in Iran. Arch Iran Med (2002) 5:184–90

[59] UNODC. Epidemiology of Drug Use in Iran. Available from: http://www.unodc.org/iran/en/epidemiology.html

[60] Jafari S Movaghar A Baharlou S Spittal P Craib K Trends of substance use in Southern Iran: a qualitative study. Internet J Epidemiol (2007) 6(1). Available from: http://ispub.com/IJE/6/1/7802

[61] Rasouli MR Nouri M Zarei MR Saadat S Rahimi-Movaghar V Comparison of road traffic fatalities and injuries in Iran with other countries. Chin J Traumatol (2008) 11(3):131–4 18507940 10.1016/s1008-1275(08)60028-0

[62] Bahadorimonfared A Soori H Mehrabi Y Delpisheh A Esmaili A Salehi M Trends of fatal road traffic injuries in Iran (2004–2011). PLoS One (2013) 8(5):e65198 10.1371/journal.pone.0065198 23724132 PMC3665536

[63] Lemoine P Ohayon M Abuse of psychotropic drugs during driving. Encephale (1996) 22(1):1–6 8681870

[64] Goullé JP Verstraete A Boulu R Costentin J Foucher JP Raes E Illicit drugs, medications and traffic accidents. Ann Pharm Fr (2008) 66(4):196–205 10.1016/j.pharma.2008.06.002 18847565

[65] Zakrajsek JS Shope JT Greenspan AI Wang J Bingham CR Simons-Morton BG Effectiveness of a brief parent-directed teen driver safety intervention (Checkpoints) delivered by driver education instructors. J Adolesc Health (2013) 53(1):27–33 10.1016/j.jadohealth.2012.12.010 23481298 PMC4147835

[66] Zakrajsek JS Shope JT Ouimet MC Wang J Simons-Morton BG Efficacy of a brief group parent-teen intervention in driver education to reduce teenage driver injury risk: a pilot study. Fam Community Health (2009) 32s2):175–88 10.1097/FCH.0b013e318199482c 19305216 PMC2747635

[67] Albery IP Strang J Gossop M Griffiths P Illicit drugs and driving: prevalence, beliefs and accident involvement among a cohort of current out-of-treatment drug users. Drug Alcohol Depend (2000) 58:197–204 10.1016/S0376-8716(99)00101-5 10669072

